# Cyclin-dependent kinase 7/9 inhibitor SNS-032 induces apoptosis in diffuse large B-cell lymphoma cells

**DOI:** 10.1080/15384047.2022.2055421

**Published:** 2022-03-25

**Authors:** Liling Jiang, Chuangyu Wen, Huan Zhou, Aochu Liu, Haichuan Zhang, Xinmei Chen, Wa Ding, Jinbao Liu, Xianping Shi

**Affiliations:** a The Sixth Affiliated Hospital of Guangzhou Medical University, Qingyuan People’s Hospital; Guangzhou Municipal and Guangdong Provincial Key Laboratory of Protein Modification and Degradatio, State Key Laboratory of Respiratory Disease; Affiliated Cancer Hospital of Guangzhou Medical University; School of Basic Medical Sciences, Guangzhou Medical University, Guangzhou P.R. China; b Department of Obstetrics and Gynaecology, Dongguan Affiliated Hospital, Southern Medical University, Dongguan, P.R. China

**Keywords:** SNS-032, apoptosis, diffuse large B-cell lymphoma, chemotherapy, cyclin-dependent kinase 7/9

## Abstract

Approximately 40% of patients with diffuse large B-cell lymphoma (DLBCL) are refractory or relapse to standard chemotherapy, and most of them are activated B cell-like DLBCLs (ABC-DLBCL) and germinal center B cell-like DLBCLs (GCB-DLBCL). SNS-032, a novel and selective CDK7/9 inhibitor, that the first phase clinical trials approved by US FDA for cancer treatment have been completed. In this study, we investigated the anti-tumor effect of SNS-032 in ABC- and GCB-DLBCL subtypes. We report that SNS-032 induced growth inhibition and cell apoptosis in both DLBCL cells in vitro, and inhibited the growth of both DLBCL xenografts in nude mice. Mechanistically, SNS-032 inhibited RNA polymerase II, which led to transcriptional-dependent suppression of NF-κB signaling pathway and its downstream targets involved in cell survival; SNS-032 also downregulates BCL-2 and c-MYC in both mRNA and protein levels. Significantly, these findings provide pre-clinical evidence for application of targeting the CDK7/9 in DLBCL.

## Introduction

Diffuse large B-cell lymphoma (DLBCL), the most common form of non-Hodgkin’s lymphoma (NHL), accounts for approximately 24%–30% of the total incidence of NHL.^1,2^ DLBCL has been divided into three subcategories according to its gene profiling patterns which are ABC-DLBCLs, GCB-DLBCLs and PMBCLs.^3,4^ The heterogeneity leads to diverse outcomes and difficulty in the application of a standard treatment for the all three subtypes. In general, at least 30% of patients who initially responded to frontline R-CHOP (rituximab, cyclophosphamide, doxorubicin, vincristine, and prednisone) chemo-immunotherapy were relapsed and died of the aggressive tumors.^5,6^ The rapid appearance of multidrug resistance results in the early demise of a considerable proportion of DLBCL patients. Hence, it is urgent to find more effective drugs and develop more innovative treatments for DLBCL.

It has been noticed that the ABC-DLBCL subtype has the worst prognosis and the most insensitive to current treatment regimens among the three subtypes.^7^ In contrast with GCB-DLBCLs (76%) and PMBCLs (64%), the patients with ABC-DLBCLs (16%) are inclined to poorer 5-y survival rate.^8^ ABC-DLBCL is characterized by activated NF-κB,^9,10^ which, after released from IκBα, tends to translocate from cytoplasm into the nucleus. NF-κB regulates cell survival and death by exerting the transcription of growth factors such as cell cycle-related genes (Cyclin D1, P53, and P21)^11^ and anti-apoptotic genes (BCL-2, MCL-1, XIAP, IAP-1, IAP-2, BFL-1, BCL-xl, and Survivin),^12,13^ thus playing a prominent role in the development of ABC-DLBCLs. GCB-DLBCLs, with the highest curability, which carrying the translocation of *BCL-2* and *c-MYC* genes, resulting in constitutive activation of both genes.^14,15^ Consequently, repression of NF-κB, c-MYC, and BCL-2 expression have been intensely investigated as potential therapeutic strategy in DLBCL patients.

SNS-032, also known as BMS-387032, is a novel selective inhibitor of CDK7 and CDK9. SNS-032 blocks the CDK7/9 mediated-phosphorylation of Ser2 and Ser5 at the C-terminal domain of RNA polymerase II (RNA pol II), thereby inhibiting transcriptional initiation and elongation.^16,17^ According to the report, blocking RNA polymerase II is effectively inhibited the proliferation of DLBCL cells.^18^ So we consided that targeting CDK7/9 should be a significant strategy to treatment DLBCL. The phase I clinical trial of SNS-032 in patients with solid tumors and advanced B-lymphoid malignancies have been completed, the efficacy and toxicity have been evaluated.^19^ Also, many researches shown that SNS-032 have a strong cytotoxic effect on a broad range of human neoplastic cells such as chronic lymphocytic leukemia (CLL), acute myeloid leukemia (AML), mantle cell lymphoma (MCL), chronic myeloid leukemia (CML) and other solid tumors.^20–23^ However, the cytotoxic effect of SNS-032 in DLBCL remains unclear; thus, we expect to evaluate the effect of targeting the CDK7/9 by SNS-032 in DLBCL cells, to explore the significance of CDK7 as a target in DLBCL therapy.

In this study, we explored the antineoplastic activity of SNS-032 in cell culture and mouse xenograft model of DLBCL cells. Obviously, SNS-032 displays significant anti-tumor effects in both GCB- and ABC-DLBCL cells and xenografts.

## Materials and methods

### Reagents

SNS-032 was purchased from Selleck Chemicals (Shanghai, China) and dissolved in dimethyl sulfoxide (DMSO) at a stock concentration of 20 mM and stored at −20°C. Annexin V and propidium iodide (PI) were obtained from Sigma-Aldrich (St. Louis, MO, USA). The Cat number of antibodies were supplied in supplementary Table S1.

### Cell culture

The DLBCL cell lines SU-DHL-4, OCI-LY-1, OCI-LY-19 (GCB-DLBCL) and SU-DHL-2 (ABC-DLBCL) cells were purchased from ATCC and cultured with RPMI 1640 medium (HyClone; GE Healthcare Life Sciences, Logan, UT, USA) supplemented with 10% inactived fetal bovine serum (Gibco; Thermo Fisher Scientific, Inc., Waltham, MA, USA), 100 unit/ml penicillin, and 10 μg/ml streptomycin (Gibco; Thermo Fisher Scientific, Inc., Waltham, MA, USA) in a humidified atmosphere of 5% CO_2_ at 37°C.

### Cell viability assay

MTS assay (CellTiter 96Aqueous One Solution reagent; Promega Co., Ltd. Shanghai, China) was performed to detect cell viability. 2 × 10^5^/ml cells in 100 μl were incubated with rising concentrations of SNS-032 for 48 hours. The control group supplemented DMSO for the same volume as the highest concentration of SNS-032 but limited in 0.1%v/v. Then, 20 μl MTS was added and detected with a 96-well plate reader at wavelength 490 nm after another 4 hours incubated.

### Cell counting assay

Increasing doses of SNS-032 added to 24-well plates which seeded 2 × 10^5^ cells (SU-DHL-4 and SU-DHL-2) for indicated duration, after that, under the light microscope, 0.4% trypan blue (Sigma-Aldrich; Merck KGaA, Darmstadt, Germany) was stained for detect the number of cells including live and dead.

### Measurement of apoptosis by flow cytometry

Apoptosis was performd by flow cytometry using Annexin V/PI double staining.^24^ Cells were incubated with indicated concentration of SNS-032, collected and washed once with PBS buffer (Sungene biotech; Tianjin, China), then incubated in working buffer (500 μl binding buffer with 5 μl Annexin V) for 15 minutes in dark, followed with 5 μl PI (Sungene biotech; Tianjin, China) addition, apoptotic cells were determined by Beckman flow cytometry and the resulting data were analyzed by CytExpert software.

### Cell cycle analysis by flow cytometry

After exposure to various durations of SNS-032 for a fixed dose, cells were collected and fixed overnight in 66% cold ethanol at 4°C. The cells were then washed twice in cold PBS and labeled with propidium iodide (Sigma-Aldrich; Merck KGaA, Darmstadt, Germany). Cell cycle distribution was determined by using BD FACScanto II flow cytometry analyzer equipped with BD FACSDiva software.

### Western blot analysis

SNS-032-treated cells were pelleted by centrifugation and rinsed with PBS. Whole cell lysates were prepared in RIPA buffer (1× PBS, 1% NP-40, 0.5% sodium deoxycholate, 0.1% SDS) supplemented with 10 mM b-glycerophosphate, 1 mM sodium orthovanadate, 10 mM NaF, 1 mM phenylmethylsulfonyl fluoride (PMSF), and 1× Roche Protease Inhibitor Cocktail (Roche, Indianapolis, IN). Western blotting was performed as we previously described.^24^

### RNA isolation and real-time quantitative polymerase chain reaction

Total RNA was isolated using Trizol reagent from 5 × 10^6^ cells (Invitrogen/Thermo Fisher Scientific, Inc.). cDNA was synthesized using the PrimeScript RT reagent Kit (TaKaRa Biotechnology Co., Ltd. Dalian, China), then five times dilution cDNA was used for real-time PCR with the SYBR Premix Ex Taq II Kit (TaKaRa Biotechnology Co., Ltd). Each PCR reaction was carried out in Bio-Rad CFX96 Real-time PCR system (Bio-Rad Laboratories, Inc., Hercules, CA, USA) within a 20 μl volume in 200 μl tubes. The specific primers for real-time PCR are shown in supplementary Table S2.

### Nude mouse xenograft model

Nude Balb/c mice were bred at the animal facility of Guangzhou medical university. The mice were housed in barrier facilities with 12 hours’ light dark cycle, with food and water available ad libitum. Mixture of 2 × 10^7^ of DLBCL cells with matrigel (BD Biosciences; Becton, Dickinson and Company, Franklin, Lakes, NJ, USA) were inoculated subcutaneously on the flanks of 5-week-old male nude mice. When the average tumor size reached about 50 mm^3^, mice were treated with either vehicle (10% DMSO, 10% cremophor, and 80% PBS) or SNS-032 (9 mg/kg of body weight) every day for totally 8 d. Tumors were measured every day by calipers. Tumor volumes were calculated by the following formula: a^2^ × b × 0.4, where a is the smallest diameter and b is the diameter perpendicular to a. The body weight was monitored as indicators of general health. The animals were then euthanized, and tumor xenografts were immediately removed, weighed, stored, and fixed. All animal studies were conducted with the approval of the Guangzhou medical university Institutional Animal Care and Use Committee.

### Immunohistochemical staining

Formalin-fixed xenografts were embedded in paraffin and sliced with 4 μm. Sections were immunostained using the MaxVision kit (Maixin Biol, ML, USA) according to the manufacturer’s instructions. Immunohistochemical staining was performed as we previously described.^24^

### Statistical analysis

All experiments were performed in triplicates at least, and results are expressed as Mean ± SD where applicable. GraphPad Prism 6.02 software (GraphPad Software, San Diego, CA) was used for statistical analysis. Comparisons between two groups involved two-sided Student’s t test, and comparisons among multiple groups involved one-way ANOVA with post hoc intergroup comparison by the Tukey test. P < .05 was considered statistically significant.

## Results

### SNS-032 inhibits cell proliferation in both GCB-DLBCL and ABC-DLBCL cell lines

**Figure 1.**
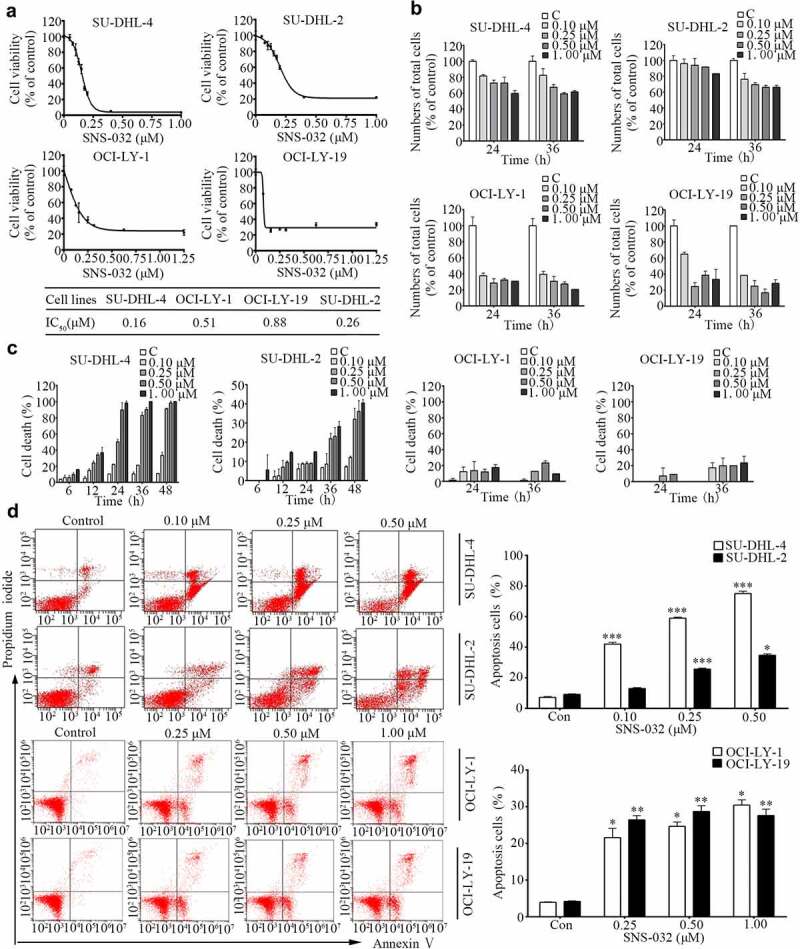
SNS-032 inhibits cell growth and induces apoptosis in both GCB- and ABC-DLBCL cells. (a) SNS-032 suppresses cell viability of SU-DHL-4, SU-DHL-2, OCI-LY-1 and OCI-LY-197 cells. Cells were cultured with escalating concentrations (0.1–1.25 μM) of SNS-032 for 48 hours. Cell viability was then examined by the MTS assay. Mean ± SD (N = 3). (b) SNS-032 induces proliferation inhibition of both GCB- and ABC-DLBCL cells. SU-DHL-4, SU-DHL-2, OCI-LY-1 and OCI-LY-19 cells were treated with various concentrations of SNS-032 (0.1–1 μM), then trypan blue exclusion staining was performed to detect total cell number. Mean ± SD (N = 3). (c) SNS-032 induces cell death in both GCB- and ABC-DLBCL cells. SU-DHL-4, SU-DHL-2, OCI-LY-1 and OCI-LY-19 cells were treated with SNS-032 (0.1–1 μM) for indicated time periods (6–48 hours). The death cell number was measured by trypan blue exclusion staining. Mean ± SD (N = 3). (d) SNS-032 induces apoptosis in GCB- and ABC-DLBCL cells. SU-DHL-4, SU-DHL-2, OCI-LY-1 and OCI-LY-19 cells were treated with indicated concentrations (0.1–1 μM) of SNS-032 for 36 hours and cell apoptosis was detected by Annexin V-FITC /PI double staining with flow cytometry. The percentage of cell apoptosis was summarized (right). Mean ± SD (N = 3). Representative images were shown. *P < .05, **P < .01, ***P < .0001, versus control group.

We investigated the toxic effect of SNS-032 on the R-CHOP sensitive GCB-DLBCL (SU-DHL-4, OCI-LY-1, and OCI-LY-19) and insensitive ABC-DLBCL (SU-DHL-2) cell lines, these four cells incubating with various concentrations of SNS-032 for 48 hours, followed by MTS assay. As shown in Figure 1(a), SNS-032 was potent to all of the DLBCL cell lines, with IC_50_ values of 0.16 μM, 0.51 μM, 0.88 μM, and 0.26 μM, respectively.

We next measured the inhibitory effect of SNS-032 on GCB- and ABC-DLBCL cell lines in a kinetic experiment by a trypan blue exclusion assay. We observed a time- and dose-dependent decrease in total cell proportion of all the four cell lines with SNS-032 incubation (Figure 1(b)).

### SNS-032 induces cytotoxicity in both GCB- and ABC-DLBCL cell lines

To confirm the cell death-inducing activity of SNS-032 in GCB- and ABC-DLBCL cell lines, cells of SU-DHL-4, OCI-LY-1, OCI-LY-19 and SU-DHL-2 were treated with escalating concentrations of SNS-032, followed by Annexin V/PI staining detected with flow cytometry analysis or by trypan blue staining analyzed by cytometry. Increased number of trypan blue-positive and AnnexinV/PI-positive cells were detected in all the four cell lines in a dose-dependent manner (Figure 1(c,d)).

### SNS-032 induces caspase activation in both GCB- and ABC-DLBCL cell lines

**Figure 2.**
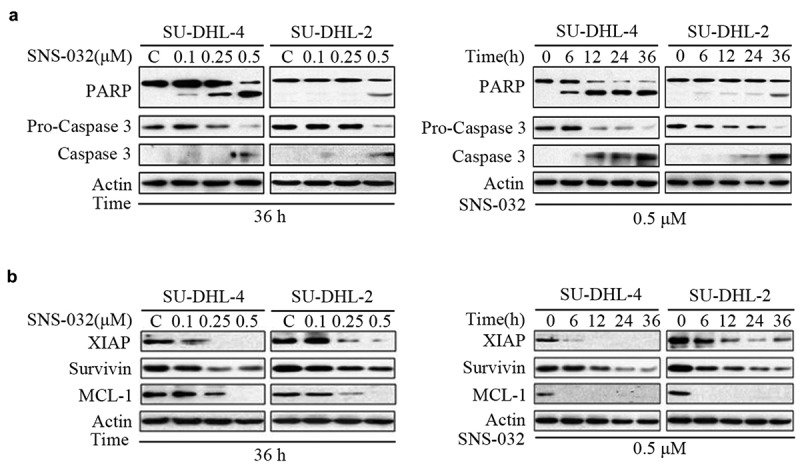
SNS-032-induced apoptosis is caspase activated and anti-apoptotic proteins decreased in both GCB- and ABC-DLBCL cells. (a) SNS-032 induces PARP cleavage and Caspase 3 activation in SU-DHL-4 and SU-DHL-2 cells. Cells were dose- and time-dependently treated with SNS-032, PARP and Caspase 3 cleavage were analyzed by Western blots. Actin was used as a loading control. C: control. (b) The expression of anti-apoptotic proteins reduced with SNS-032 treatment in SU-DHL-4 and SU-DHL-2 cells. Cells were dose (0.1–0.5 μM)- and time (6–36 hours)-dependently treated with SNS-032. The apoptosis-associated proteins Mcl-1, XIAP, Survivin were analyzed by Western blot.

To investigate or confirm that SNS-032 has apoptosis-inducing activity, SU-DHL-4 and SU-DHL-2 cell lines were incubated with SNS-032, then analyzed the levels of apoptosis-associated proteins. Cleaved PARP was increased in a dose- and time-dependent manner (Figure 2(a)). Consistently, SNS-032 treatment decreased expression of precursor forms of Caspase 3 and increased expression of active forms of Caspase 3, consistent with the model of PARP cleavage (Figure 2(a)). The data supported the conclusion that SNS-032 treatment activates caspase pathway and induces apoptosis in these DLBCL cells.

Moreover, other apoptosis-related proteins were also measured. Western blot analysis in Figure 2(b) revealed a substantial decrease in levels of several anti-apoptotic proteins, including MCL-1, XIAP and Survivin in a concentration- and time-dependent manner in both SU-DHL-4 and SU-DHL-2 cell lines.

### SNS-032 down-regulates proteins and mRNA levels of NF-κB, BCL-2, and c-MYC

**Figure 3.**
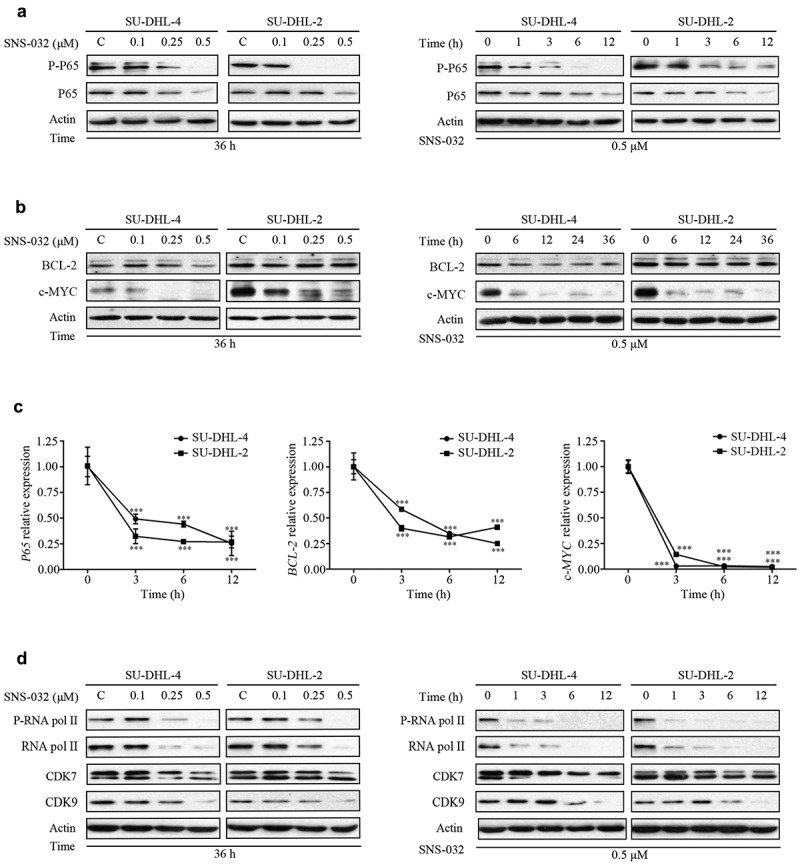
SNS-032 induces the downregulation of NF-κB, c-MYC and BCL-2 in ABC- and GCB-DLBCL cells. (a) SU-DHL-4 and SU-DHL-2 were dose (0.1–0.5 μM)- and time (1–12 hours)-dependently treated with SNS-032, the phosphorylated and total protein levels of P65 in both SU-DHL-2 and SU-DHL-4 cells were analyzed by Western blot. (b) SU-DHL-4 and SU-DHL-2 were dose (0.1–0.5 μM)- and time (6–36 hours)-dependently treated with SNS-032, the protein levels of BCL-2, c-MYC in both SU-DHL-2 and SU-DHL-4 cells were analyzed by Western blot. (c) SNS-032 down-regulates the mRNA expression of *P65, BCL-2* and *c-MYC* time-dependently. SU-DHL-4 and SU-DHL-2 cells were exposed to 0.5 µM SNS-032 for indicated time, quantitative RT-PCR analysis of mRNA level of *P65, BCL-2* and *c-MYC* was conducted. Their expression levels relative to the control were calculated. Mean ± SD (N = 3). ***P < .0001, versus control. (d) SNS-032 inhibits cellular activity of RNA pol II. SU-DHL-4 and SU-DHL-2 were dose (0.1–0.5 μM)- and time (1–12 hours)-dependently treated with SNS-032, the phosphorylated and total protein levels of RNA pol II, CDK7 and CDK 9 were analyzed by Western blot.

As mentioned, the ABC-DLBCL subtype is characterized by constitutive activation of NF-κB pathway.^9^ Inhibitor of NF-κB signaling is toxic to ABC-DLBCL cells,^25,26^ To investigate whether SNS-032 treatment inhibits NF-κB signaling in ABC- and GCB-DLBCL cell lines, SU-DHL-4, and SU-DHL-2 cells were maintained with various doses of SNS-032. We found that the levels of both phosphorylated and total P65 were decreased in a dose- and time-dependent fashion in both cell lines (Figure 3(a)). It is known that translocation of *BCL-2* and *c-MYC* genes leads to constitutive activation of both proteins, a key feature of GCB-DLBCL subtype.^14^ We found that SNS-032 treatment attenuated the protein level of c-MYC with a dose- and time-dependent manner in both SU-DHL-4 and SU-DHL-2 cell lines. However, the protein level of BCL-2 was decreased in SU-DHL-4 cells, which is GCB-DLBCL subtype, but not in SU-DHL-2 cells (Figure 3(b)). Furthermore, qRT-PCR assay revealed that SNS-032 decreased the mRNA levels of P65, BCL-2, and c-MYC in a time-dependent manner in both SU-DHL-4 and SU-DHL-2 cell lines (Figure 3(c)). Therefore, SNS-032 treatment led to NF-κB, BCL-2, and c-MYC at both the transcription and protein level in most of DLBCL cells. SNS-032, as a selective inhibitor of CDK7 and 9, consequently disables RNA pol II and gene transcription.^16^ Indeed, exposing SU-DHL-4 and SU-DHL-2 cell lines to increasing concentrations of SNS-032 or a fixed concentration of SNS-032 for various durations led to a decrease in phosphorylated RNA pol II at Ser2 and Ser5 and total protein (Figure 3(d)). These findings are in accordance with the mechanism of SNS-032 inhibiting the activity of RNA pol II by competing with ATP-binding pocket to CDK7 and CDK9.^21^

### SNS-032 induces G1 phase arrest in both GCB- and ABC-DLBCL cell lines

**Figure 4.**
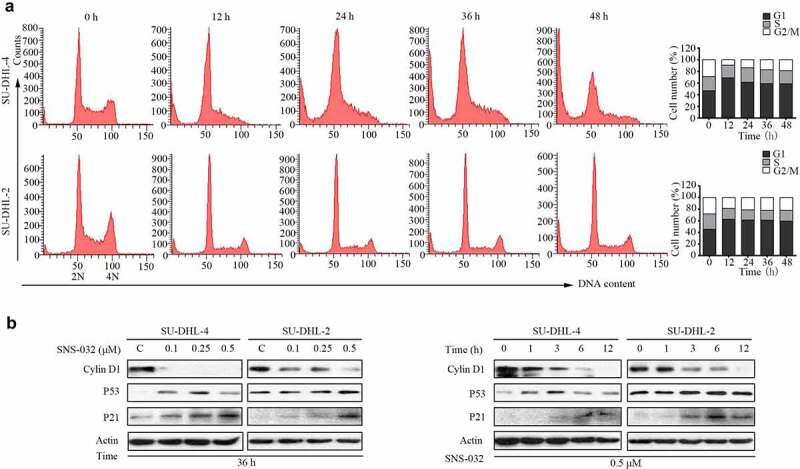
SNS-032 affects the distribution of cell cycle in both GCB- and ABC-DLBCL cells. (a) SNS-032 induces cell arrest in G1 phases. SU-DHL-4 cells were treated with 0.25 µM SNS-032 while SU-DHL-2 cells were treated with 0.5 µM SNS-032 for indicated time (12–48 hours), then cell cycle was analyzed by flow cytometry. Graphs show data from a representative experiment. (b) SNS-032 affects the expression of G1 checkpoint-related proteins. SU-DHL-4 and SU-DHL-2 cells were dose- and time-dependently treated with SNS-032, then Cyclin D1, P53 and P21 proteins were detected by Western blot.

We have found that SNS-032 potently inhibits the proliferation of two types of DLBCL cells (Figure 1(a,b)). We then investigated whether SNS-032 treatment has an effect on cell cycle distribution. Again, SU-DHL-4 and SU-DHL-2 cell lines were exposed to escalating durations of SNS-032 with an indicated concentration. Flow cytometric analysis (Figure 4(a)) revealed that the increased levels of G1 population is associated with the decreased levels of G2/M population, indicating growth arrest in G1 phase in these two cell lines. Accompanied with that, a sub-G1 apoptotic population also appeared in both cell lines which further confirmed that SNS-032 induced apoptosis.

To further elucidate the mechanism of SNS-032-induced cell cycle arrest in G1 phase, we evaluated the expression of G1 checkpoint-related proteins, such as Cyclin D1, P53, and P21. The results clearly revealed that the addition of SNS-032 decreased the level of Cyclin D1 protein and increased the levels of P21 proteins in both SU-DHL-4 and SU-DHL-2 cells, and P53 was obviously accumulated in SU-DHL-4 while slightly accumulated in SU-DHL-2 (Figure 4(b)).

### SNS-032 down-regulates the protein levels of cell growth related signaling pathway

**Figure 5.**
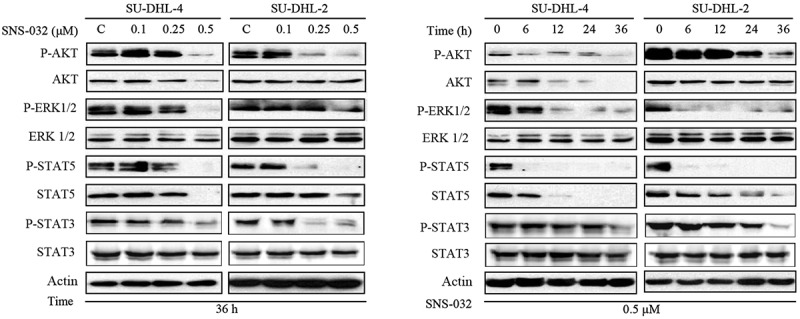
SNS-032 downregulates the expression of cell growth associated signaling pathway proteins such as ERK1/2, STAT5, STST3, and AKT. SU-DHL-4 and SU-DHL-2 cells were dose- and time-dependently treated with SNS-032, cell lysates were analyzed by Western blot.

Pathways including PI3K/AKT, MAPK/ERK, and JAK/STATs are generally regarded to elicit positive activity for tumor cell growth. We then detected the expression of core proteins in these signaling pathways with SNS-032 treatment in SU-DHL-4 and SU-DHL-2 cell lines. After SNS-032 treatment, the phosphor-AKT, phosphor-ERK1/2, phosphor-STAT3, and phosphor-STAT5 proteins were significantly reduced in a concentration- and time-dependent manner suggesting that SNS-032 restrain major cell growth signaling pathways. Also, the decrease was appeared in total protein level of AKT and STAT5 but not ERK1/2 and STAT3 (Figure 5), Combining the results shown in Figure 1(a,b), we have demonstrated a potent inhibitory effect of SNS-032 on the proliferation of DLBCL cells.

### SNS-032 inhibits the growth of GCB- and ABC-DLBCL tumor in nude mice

**Figure 6.**
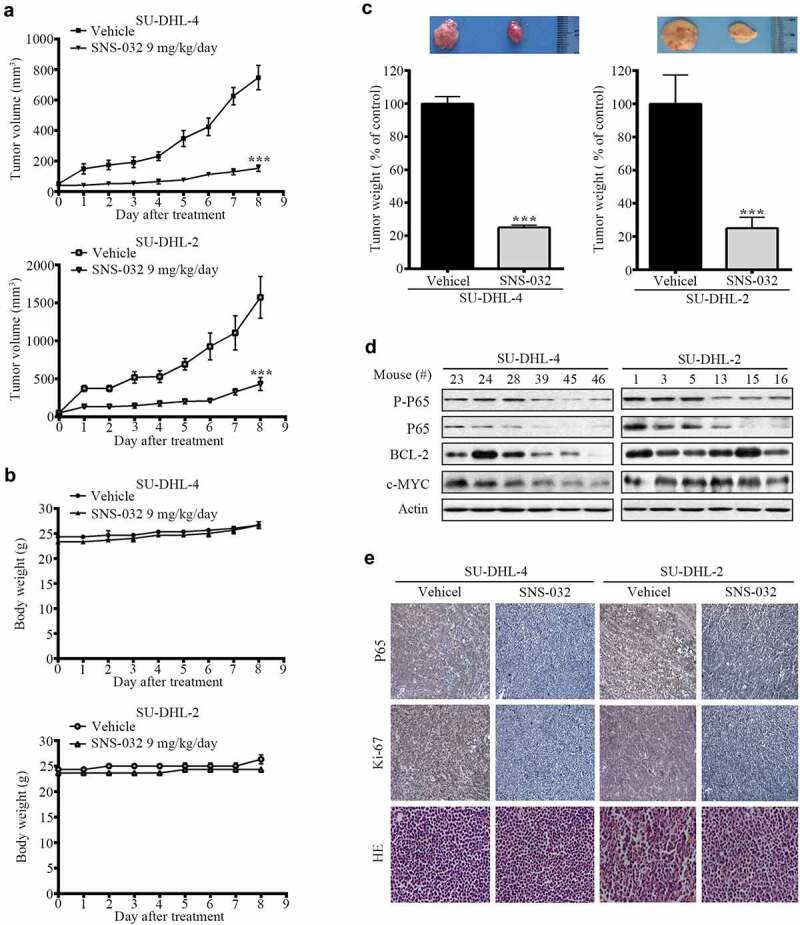
SNS-032 impairs growth of tumor cells in GCB- and ABC-DLBCL xenografted nude mice. Nude BALB/c mice were inoculated subcutaneously with SU-DHL-4 and SU-DHL-2 cells and randomly divided to vehicle- and SNS-032 (9 mg/kg/d)-treatment group. When the tumor volume reached 50mm^3^ start to treatment. (a) Tumor size was measured every day. The terminal mean tumor volumes of SU-DHL-4/vehicle, SU-DHL-4/SNS-032, SU-DHL-2/vehicle, SU-DHL-2/SNS-032 were 175 ± 18 mm^3^, 756 ± 75 mm^3^, 486 ± 36 mm^3^, 1625 ± 128 mm^3^, respectively. n = 12, ***P < .0001, versus vehicle treatment. (b) Mice weights were recorded every day after SNS-032 treatment. The terminal mean mice weights of SU-DHL-4/vehicle, SU-DHL-4/SNS-032, SU-DHL-2/vehicle, SU-DHL-2/SNS-032 were 26.2 ± 0.5 mg, 26.1 ± 0.47 mg, 26.7 ± 0.5 mg, 23 ± 0.2 mg, n = 6. (c) Tumors weights on day 8 post initial treatment were calculated and shown. Mean ±SD, n = 12. ***P < .0001. versus vehicle treatment. Western blot(d) analyzed the expression of P-p65, p65, Bcl2 and c-Myc. Immunohistochemistry(e) analysis was performed to examine p65 and Ki-67 in the tumor tissues. (SU-DHL-4 control group: #23, #24, #28; SNS-032-treated group: #39, #45, #46; SU-DHL-2 control group: #1, #3, #5; SNS-032-treated group: #13, #15, #16). All the immunostaining and Western blot were repeated in three mouse tumor tissues and the most typical images were shown.

We investigated the in vivo effects of SNS-032 with a nude mouse xenograft model. Four days after subcutaneous inoculation of SU-DHL-4 and SU-DHL-2 cell lines, the mice were randomized to receive treatments with either vehicle or SNS-032 (9 mg/kg/d) for approximately 8 d. We found that SNS-032 treatment significantly suppressed the growth of both GCB- and ABC-DLBCL xenografts (Figure 6(a)); while body weights of the mice remained relatively stable in each group (Figure 6(b)), motor activity and feeding behavior were all normal. The mean weights of tumors were significantly reduced in SNS-032-treated group compared to the vehicle-treated group (Figure 6(c)). Immunohistochemical analysis revealed that SNS-032 treatment significantly induced growth arrest (Ki-67) (Figure 6(e)). SNS-032 treatment caused inhibition of NF-κB in xenografts of both subtypes, evidented by decreased protein level of total and phosphorylated P65 compared with vehicle (Figure 6(d)). However, this drug only inhibits BCL-2 and c-MYC expression in SU-DHL-4, but not SU-DHL-2 tumors (Figure 6(d)). In conclude, the results illustrated that SNS-032 suppresses the growth of both GCB- and ABC-DLBCL tumors in vivo.

## Discussion

Although more than half of DLBCL patients could be cured by standard R-CHOP regimens, relapsed/refractory DLBCL remains a major cause of morbidity and mortality. NF-κB over-expression and excessive activation were found to be the independent prognostic marker for poor survival and drug resistance in DLBCL.^27,28^ Particularly, ABC subtype of DLBCL, as distinguished by increased dependence on the NF-κB signaling pathway, tends to be refractory to chemotherapy. It has been shown that GCB-DLBCL cells, featured by *BCL-2/c-MYC* double-hitted translocation, are response favorably to chemotherapy but there are still small percentage are resistant.

There are several emerging agents have shown promising effects in chemo-resistant DLBCLs. For example, the bruton tyrosine kinase (BTK) inhibitors ibrutinib which elicts limited single-agent activity in DLBCLs but show a good therapeutic effect when combining with other immunotherapy agents, such as lenalidomide and rituximab.^29^ The promising therapy, chimeric antigen receptor (CAR) T-cell therapy approved by the U.S. FDA in 2017, changed the treatment landscape of DLBCL. CAR T cells provide an efficacious treatment option for chemo-resistant patients that ineligible for autologous hematopoietic stem cell transplantation (AHCT). However, treatment with CAR T cells produces nonnegligible toxicities, as cytokine-release syndrome and immune effector cell-associated neurotoxicity syndrome, which limits its application.^30^ Also, several anti-cancer agents that targeting NF-κB and BCL-2 have been investigated in DLBCL. For example, the 20S proteasome inhibitor bortezomib, inhibits canonical NF-κB signaling pathway by accumulation of IκBα which is an inhibitor of NF-κB. A preclinical/clinical phase II study demonstrated that bortezomib can selectively sensitize patients with relapsed/refractory ABC-DLBCL, but not patients with relapsed/refractory GCB-DLBCL, to chemotherapy.^31^ Furthermore, bortezomib has limited single-agent activity in relapsed/refractory DLBCL.^32^ Another example is small-molecule BH3 mimetics which are BCL2 inhibitors, including ABT-737, ABT 263, and GX15-070. Recent studies have suggested that MCL-1 mainly contributes to chemotherapy resistance in ABC-DLBCL; however, ABT-737 and ABT 263 were unable to downregulate MCL-1.^33,34^ GX15-070, a pan-BCL-2 inhibitor, inactivates MCL-1 but causes dose-dependent thrombocytopenia.^35^ Therefore, seeking for the novel small-molecule inhibitors that have efficacy to both ABC- and GCB-DLBCL cells or potential to be combined with chemotherapy should be an alternative strategy to reverse relapsed/refractory for DLBCL patients.

SNS-032, which is a potent inhibitor of CDK7 and 9 and blocks transcription enabling-phosphorylation of RNA pol II, has antitumor activities in a broad range of human cancer cells. However, the effect of SNS-032 on NF-κB, BCL-2, and c-MYC in DLBCL has not been reported. The results of our current study suggest that SNS-032 dramatically induces cytotoxicity in DLBCL cells including both ABC- and GCB-DLBCL subtypes. In the cell culture experiments, SNS-032 decreased cell viability and cell proliferation in both ABC- and GCB-DLBCL cell lines. The IC_50_ of SNS-032 in the two types of DLBCL cells are less than one micromole. According to the results there was no distinct difference between ABC and GCB DLBCL cell types. Also, SNS-032 elicits apoptosis in ABC and GCB DLBCL cells in a time- and dose-dependent manner, consisting with downregulated Caspase3 and increased stain of Annexin Ⅴ/PI. We also found that SNS-032 downregulated anti-apoptosis proteins including MCL-1, which suggests its potential to be combined with a BCL-2 inhibitor. Echo the effects of SNS-032 in other model systems, the total and phosphorylated RNA pol II was inhibited. The key overexpressed proteins in two types DLBCL, c-MYC, and P65 are downregulated in both mRNA and protein levels. While a declined BCL-2 protein was absent in SU-DHL-2 (ABC-DLBCL) and slightly seen in SU-DHL-4 (GCB-DLBCL), but BCL-2 mRNA was significantly declined in both cell lines, owing to the different mechanisms of BCL-2 regulation between GCB and ABC DLBCL.^36,37^ The result further shown that SNS-032 induced cell cycle arrest in DLBCL cells was associated with decreased Cyclin D1, as well as increasing levels of P53 and P21.

Together, our results illustrate that SNS-032 displays a significant anti-tumor effect in GCB and ABC subtypes of DLBCL cells both in vitro and in vivo. Transcriptional inhibition-induced suppression of NF-κB and c-MYC may contribute to SNS-032-induced cytotoxicity in ABC and GCB DLBCL cells. These findings suggest for the first time that inhibiting transcription with SNS-032 targeting CDK7/9 is toxic to DLBCL cells and indicates that CDK7/9 is feasible as a therapeutic target of DLBCL.

## Supplementary Material

Supplemental Material
