# Supplementary material for: Cyclin-dependent kinase 7/9 inhibitor SNS-032 induces apoptosis in diffuse large B-cell lymphoma cells
Source: Cancer Biol Ther. 2022 Mar 25;23(1):319–27. doi: 10.1080/15384047.2022.2055421 (PMC8959513; doi:10.1080/15384047.2022.2055421)
Supplement: Supplemental Material [file KCBT_A_2055421_SM5061.docx]

Supplementary Table 1. The Cat. Number of antibodys.

| p65 | Cell Signaling Technology (8242s) |
| --- | --- |
| phospho-p65 at Ser536 | Bioworld Technology (bs4138) |
| RNA polymerase II (pol II) | Cell Signaling Technology (2629s) |
| phospho-RNA Pol II at Ser2 and Ser5 | Cell Signaling Technology (4735s) |
| cyclin-dependent kinase 7 (CDK7) | Cell Signaling Technology (2916s) |
| cyclin-dependent kinase 9 (CDK9) | Cell Signaling Technology (2316s) |
| Caspase-3 | Cell Signaling Technology (9662S) |
| cleaved-Caspase-3 | Cell Signaling Technology (9579s) |
| Mcl-1 | Proteintech (16225-I-Ap) |
| XIAP | Cell Signaling Technology (2042S) |
| Survivin | Cell Signaling Technology (2808S) |
| Cyclin D1 | Cell Signaling Technology (2926p) |
| p21 | Cell Signaling Technology (2947p) |
| AKT | Cell Signaling Technology (9272S) |
| phospho-AKT at Ser473 | Cell Signaling Technology (9271S) |
| Erk1/2 | Cell Signaling Technology (9102S) |
| phospho-Erk1/2 at T202/Y204 | Cell Signaling Technology (4370S) |
| STAT5 | Cell Signaling Technology (9363S) |
| phospho-STAT5 at Y694/Y699 | Merck Milipore (50095) |
| Bcl-2 | Cell Signaling Technology (15071S) |
| PARP | Cell Signaling Technology (9542S) |
| p53 | Bioworld Technology (bs1913) |
| Ki-67 | Cell Signaling Technology (9449S) |
| Actin | Proteintech (205365-I-AP) |
| c-Myc | Cell Signaling Technology (18583S) |
| Anti-mouse IgG-HRP | Merck Milipore (12-349) |
| Anti-rabbit IgG-HRP | Merck Milipore (AP132P) |

Supplementary Table 2. The specific primers for real-time PCR.

| p65-Forward | 5′- ACCTCGACGCATTGCTGTG -3′ |
| --- | --- |
| p65-Reverse | 5′- CTGGCTGATCTGCCCAGAAG -3′ |
| Bcl-2-Forward | 5′- AACATCGCCCTGTGGATGAC -3′ |
| Bcl-2-Reverse | 5′- AGAGTCTTCAGAGACAGCCAGGAG -3′ |
| c-MYC-Forward | 5′- GGA GGC TAT TCT GCC CAT TTG -3′ |
| c-MYC-Reverse | 5′- CGA GGT CAT AGT TCC TGT TGG TG -3′ |
| GAPDH-Forward | 5′- CCT GAC CTG CCG TCT AGA AAA -3′ |
| GAPDH-Reverse | 5′- TGG GTG TCG CTG TTG AAG TC -3′ |
